# S100A4 targets PPP1CA/IL-17 to inhibit the senescence of sheep endometrial epithelial cells

**DOI:** 10.3389/fvets.2024.1466482

**Published:** 2024-11-27

**Authors:** Xiyao Jiao, Yaoxuan Jiao, Jingwen Cui, Haorui Zhang, Xiangyun Li, Zhili Chu, Xinglong Wu

**Affiliations:** ^1^College of Animal Science and Technology, Hebei Technology Innovation Center of Cattle and Sheep Embryo, Hebei Agricultural University, Baoding, China; ^2^School of Basic Medical Sciences, Xinxiang Medical University, Xinxiang, China; ^3^Henan International Joint Laboratory of Immunity and Targeted Therapy for Liver-Intestinal Tumors, Xinxiang Medical University, Xinxiang, China

**Keywords:** Cell senescence, endometrial epithelial cells, S100A4, PPP1CA, IL-17

## Abstract

**Background:**

Gonadotropin-releasing hormone (GnRH) is commonly used in animal reproduction and production, but it was previously reported that GnRH decreases the embryo implantation rate during artificial insemination or embryo transfer in sheep. In addition to the finding that GnRH can target S100A4 to inhibit endometrial epithelial cells proliferation, it was also found that endometrial cells were in poor condition and experienced cell death in S100A4 knockout mice, but the mechanism is unclear.

**Methods:**

The protein PPP1CA, which interacts with S100A4, was detected by immunoprecipitation-mass spectrometry of overexpression and knockdown of S100A4 and PPP1CA. The effect of S100A4 and PPP1CA on cell senescence was detected by Galactosidase staining. To further reveal the mechanism effect of S100A4 and PPP1CA on cell senescence, transcriptome sequencing was conducted. Additionally, *in vivo* experiments were performed to assess PPP1CA protein expression in the endometrial tissue of S100A4 knockout mice.

**Results:**

S100A4 inhibited cell senescence by activating PPP1CA, while PPP1CA overexpression suppressed the activation of the IL-17 signaling pathway. Inhibition of the IL-17 signaling pathway inhibited the senescence of endometrial cells.

**Conclusion:**

S100A4 can target the PPP1CA/IL-17 signaling pathway and inhibit endometrial epithelial cell senescence.

## 1 Introduction

Sheep are important livestock species worldwide that provide humans with meat, leather, and wool. Despite high breeding numbers, many traditional local breeds may not meet the efficiency demands for growth rates and meat yields. Therefore, breeding activities to improve quality are crucial for sheep farming, with artificial insemination and embryo transfer representing technologies for rapid breeding improvement. GnRH is associated with the dual activity of FSH and LH; it can promote ovulation and is widely used in assisted reproductive technology in sheep, cattle, and humans. However, previous studies have shown that GnRH administration at the time of insemination reduced pregnancy rates in Kazak ewes (1). Similarly, GNRH administration to recipient ewes also reduced the embryo transfer success rate (2). In human-assisted reproductive technology, two main protocols for the use of GnRH are the gonadotrophin-releasing hormone agonist (GnRH-a) protocol and the gonadotrophin-releasing hormone antagonist (GnRH-ant) protocol. GnRH can effectively promote ovulation, but it has not been shown to significantly improve the outcome of fresh embryo transfers (3). Neither protocol has achieved perfect results in promoting embryo implantation (4). For example, GnRH-a pretreatment seems to improve frozen–thawed embryo transfer outcomes but is associated with a higher preterm birth rate (5).

Many factors can influence embryo implantation, including the proliferative capacity of endometrial cells, cell apoptosis (6), and cell senescence (7), all of which can affect the uterine environment and further impact embryo implantation. Our previous results indicated that GnRH can reduce the expression level of S100A4 in the endometrium and suppress endometrial cell proliferation (8). However, we also found that mice with endometrial S100A4 knockout tended toward a thinner endometrium and increased endometrial cell apoptosis, suggesting that GnRH treatment might also accelerate endometrial cell aging and death. Protein phosphatase 1 catalytic subunit alpha (PPP1CA) is an alpha subunit of the PP1 complex that is known to be involved in the regulation of various cellular processes (9). Evidence suggests that PPP1CA can inhibit cell senescence (10).

This study identified PPP1CA as an interacting protein downstream of S100A4. *In vitro* experiments verified the effect of PPP1CA on the senescence of sheep endometrial cells, and *in vivo* experiments using S100A4 knockout mice revealed that the absence of S100A4 could decrease PPP1CA protein levels. Further transcriptome sequencing revealed that IL-17 is downstream of S100A4 and PPP1CA, suggesting that inhibiting IL-17 can suppress the senescence of sheep endometrial cells. These findings provide a theoretical basis for the use of GnRH in sheep embryo transfer.

## 2 Materials and methods

### 2.1 Isolation and culture of endometrial epithelial cells from sheep

The methods used to isolate and culture the cells were described in our previous study (8). Briefly, freshly excised uteri were soaked in PBS containing penicillin (100 IU/mL) and streptomycin (0.1 mg/mL) and transported to the laboratory. The uteri were washed three times with PBS containing antibiotics. Under a microscope, the endometrium was peeled off, cut into 1–2 mm^3^ pieces, and evenly spread on a cell culture dish. The dish was incubated at 37°C for 1 h to allow the tissue fragments to adhere to the dish. Then, DMEM (Cytiva) containing antibiotics and 10% FBS (fetal bovine serum, PAN-Biotech) was added. The culture mixture was returned to the incubator and maintained for 7–10 days to allow cells to migrate from the tissue edges. After this incubation period, the cells were digested and passaged for use in subsequent experiments.

### 2.2 β-galactosidase staining

A β-galactosidase staining kit was purchased from Beyotime Biotechnology. The staining procedure was performed according to the manufacturer’s instructions. The main steps involved fixing the cells with a fixative solution for 15 min, preparing the staining solution, adding it to the cell dishes, and incubating the cells in a cell culture incubator for 1 h. After removing the staining solution, the cells were washed once with PBS, and fresh PBS was added for imaging (Ts2R-FL, Nikon, Japan).

### 2.3 Y-320 treatment

Y-320 was dissolved in DMSO to prepare a concentrated solution (5 mg/mL). To prepare the working solution, 50 μL of the concentrated solution was added to a 20% SBE-*β*-CD in saline solution. The mixture was gently shaken and left to stand for a few minutes until clear. Y-320 was added to sheep endometrial cells at a dilution of 1:10,000 in the cell culture medium, resulting in a final concentration of 0.05 μg/mL (approximately 99 nM). After culturing for 48 h, *β*-galactosidase staining was performed.

### 2.4 siRNA transfection and lentiviral packaging

siRNAs were purchased from GENEWIZ Biotechnology Co., Ltd., and their sequence information is provided in Table 1. Before transfecting siRNA, sheep endometrial cells were seeded into 6-well culture plates at a density of 5 × 10^4 cells per well. The next day, non-adherent cells were discarded and fresh medium was added. To transfect the cells, 50 pmol of siRNA was mixed with 200 μL of Opti-MEM™ I Reduced Serum Medium (Cat. No. 31985070, Gibco). Separately, 5 μL of RNAiMAX (Lipofectamine™ RNAiMAX, Cat. No. 13778-075, Invitrogen) was also mixed with 200 μL of Opti-MEM. After incubating each mixture for 5 min, the solutions were combined and allowed to stand at room temperature for 15 min. The siRNA complex was then added to the cells and incubated at 37°C. The medium was not changed until the cells were harvested for further analysis. As a control, NC (Negative Control siRNA) transfections were performed in parallel.

**Table 1:** siRNA and primers sequences.

Name	Sense (5′-3′)	Antisense (5′-3′)
siPPP1CA-1	GAAGCUCAACCUGGACUCUAUTT	AUAGAGUCCAGGUUGAGCUUCTT
siPPP1CA-2	GCAAGAGACGCUACAACAUCATT	UGAUGUUGUAGCGUCUCUUGCTT
siPPP1CA-3	GCUGGCCUAUAAGAUCAAGUATT	UACUUGAUCUUAUAGGCCAGCTT
siNC	UUCUCCGAACGUGUCACGUTT	ACGUGACACGUUCGGAGAATT
GAPDH	AGGTCGGTGTGAACGGATTTG	TGTAGACCATGTAGTTGAGGTCA
IL-17	TAGCGGTAAAGACGGTGGAG	TTTACCCTTGCTGGTGCAGT

Lentiviruses overexpressing S100A4 and PPP1CA were synthesized by GeneChem Co., Ltd., with the synthesis report provided in Supplementary materials 2, 3. During lentiviral packaging, the backbone vector, along with the auxiliary vectors VSVG and PAX2, were transfected into 293 T cells at a 2: 1:1 ratio. Forty-eight hours after transfection, the cell supernatant was collected and used to infect sheep cells. The sheep cells were seeded in a 60 mm dish. Once the cells reached 60% confluency, the culture medium was discarded, and 6 mL of the lentiviral supernatant was added, along with Polybrene at a final concentration of 5 μg/mL. After 6 h of incubation, the viral medium was replaced with fresh culture medium, and the cells were further cultured. After 48–72 h, once most of the sheep cells expressed GFP, they were ready for subsequent experiments.

### 2.5 EdU staining

An EdU staining kit was purchased from Beyotime Biotechnology. The main steps of the staining protocol were as follows: EdU was diluted in cell culture medium to a final concentration of 10 μM. The original culture medium was discarded and replaced with EdU-containing medium. The cells were incubated in a cell culture incubator for 3–4 h, fixed with 4% paraformaldehyde for 15 min, and permeabilized with 0.25% Triton X-100 for 12 min. The Click reaction mixture was then prepared according to the manufacturer’s instructions and added to the cells. The cells were incubated at room temperature in the dark for 1 h, after which the reaction mixture was discarded. The cells were washed once with PBS, fresh PBS containing hoechst33342 was added, and the cells were imaged after 5 min of incubation at room temperature.

### 2.6 Transcriptome sequencing and analysis

RNA samples from sheep endometrial cells with S100A4 knockdown were obtained by transfecting siRNA targeting S100A4, following the same procedure as described in Section 2.3. After 24 and 48 h post-transfection, the medium was discarded, and the cells were washed twice with PBS. Each well of a 6-well plate was lysed using 1 mL of RNAiso Plus (Cat. No. 9109, TAKARA), and three wells of similarly treated cells were pooled into one sample.

For generating samples overexpressing PPP1CA, lentiviral vectors overexpressing PPP1CA were packaged in 293T cells, following the method described in Section 2.3. The harvested lentiviral supernatant was centrifuged, and the cells were seeded in a 60 mm dish. Once the cells reached 60% confluency, the culture medium was discarded, and 6 mL of the lentiviral supernatant was added, along with Polybrene at a final concentration of 5 μg/mL. After 6 h of incubation, the viral medium was replaced with fresh culture medium, and the cells were further cultured. Sequencing and analysis were performed by Beijing Novo Biotechnology Co., Ltd. Transcriptome sequencing was performed by Suzhou Jinweizhi Biotechnology Co., Ltd.

Differential gene expression was analyzed using the DESeq2 package, and a volcano plot was generated with log10 fold-change values on the y-axis. GO and KEGG enrichment analyses were conducted using the clusterProfiler program, while Pearson correlation heatmaps were generated using Pheatmap.

For samples with S100A4 knockdown, NC1, NC2, and NC3 represent Negative Control Sample1, Negative Control Sample2 Negative, and Control Sample3, respectively, and KD1, KD2, and KD3 represent Knock down Sample 1, Knock down Sample 2, and Knock down Sample 3, respectively. For samples overexpressing PPP1CA, Control represents the control group with empty vector, and PPP1CA represents the experimental group with overexpression.

### 2.7 Mass spectrometry and co-immunoprecipitation (Western blot)

In sheep endometrial cells overexpressing HA-tagged S100A4 (HA is the tag added when constructing the vector), 48 h after transfection, cells were washed twice with PBS. The cells were then lysed using NP-40 lysis buffer containing a protease inhibitor and incubated on ice for 30 min. The lysate was collected into a centrifuge tube and spun at 12,000 rpm for 10 min at 4°C. The supernatant was incubated with Protein A/G resin pretreated with an HA primary antibody (anti-HA, Cat. No. AE105, Abclone) at room temperature for 2 h. The resin was then washed six times with NP-40 buffer, and the supernatant was discarded after each wash by centrifuging at 500 rpm for 5 min. As a control, Protein A/G resin without the HA primary antibody was also used. The bound protein was eluted with protein loading buffer, heated at 100°C for 10 min, then cooled to room temperature. Protein samples were run SDS-PAGE on electrophoresis at 110 V for 1 h, and the gel was sent to Jin Kairui Biotechnology, Ltd. for mass spectrometry-based detection and analysis. GO enrichment analysis of the identified proteins was performed using the clusterProfiler program.

For Co-immunoprecipitation, HA-S100A4 and Flag-PPP1CA proteins were overexpressed in 293 T cells. When protein samples were collected 48 h after transfection, a small amount of the cell lysate was taken in advance for verification. Western blot analysis was performed with 12% polyacrylamide gel electrophoresis for both the co-precipitated and whole-cell proteins. Thirty micrograms of protein were loaded into each well, and the maximum volume of immunoprecipitated protein was used. Electrophoresis was carried out at 110 V for 1.5 h, followed by protein transfer onto a PVDF membrane at 300 mA on ice for 1.5 h. The membrane was blocked with 10% skimmed milk for 30 min, then rinsed with TBST for 10 min. The corresponding primary antibodies (anti-HA, Cat. No. AE105, ABclonal; anti-Flag, Cat. No. AE092, ABclonal) were added, and the membrane was incubated overnight at 4°C. The next day, the membrane was washed three times with TBST, followed by incubation with HRP-conjugated secondary antibody (HRP Conjugated AffiniPure Goat Anti-rabbit IgG (H + L), Cat. No. BA1055, Boster) at room temperature for 1 h. After washing three more times with TBST, Clarity Western ECL Substrate (Bio-Rad) was added to the membrane. The PVDF membrane was then placed in a chemiluminescence analyzer (MiniChemi® 610, SINSAGE) for imaging.

### 2.8 Immunofluorescence staining

Cells were fixed with 4% paraformaldehyde for 15 min and permeabilized with 0.25% Triton X-100 for 12 min, followed by three washes in PBS for 5 min each. The cells were blocked with 1% BSA for 30 min and then incubated overnight at 4°C with primary antibodies diluted in 1% BSA (S100A4 antibody, 1:100, sc-377059, Santa Cruz, USA; PPP1CA, A24288, Abclone, China). After three washes in PBS, the cells were incubated with fluorescent secondary antibodies (DyLight 594-conjugated AffiniPure goat anti-mouse IgG (H + L) (1:100, BA1141, Boster, China) and ABflo® 488-conjugated goat anti-rabbit IgG (H + L) (1:100, AS053, Abclone, China)) for 1 h at room temperature in the dark. After three washes in PBS, the cells were stained with PBS containing Hoechst 33342 and imaged with a confocal laser scanning microscope.

### 2.9 HE staining and immunohistochemistry

HE staining was performed as described previously (8). For immunohistochemistry, paraffin sections were deparaffinized, repaired, blocked, and then incubated overnight with a PPP1CA antibody (1:100, A24288, Abclone, China). After the samples were reacted with an HRP-conjugated goat anti-rabbit secondary antibody, color development was performed using a DAB staining kit (G1212-200 T, Servicebio, China). The sections were counterstained with hematoxylin, rinsed with tap water, mounted, and imaged under a microscope.

### 2.10 Animal breeding and analysis

To generate uterine-specific S100A4 knockout mice, C57BL/6J-S100A4em1 (flox) Cya mice (strain number CKOCMP-20198-S100a4-B6J-VA, Cyagen Biosciences, Inc.) were crossed with PR-Cre transgenic mice (model number C001035, Cyagen Biosciences, Inc.). Homozygous flox mice positive for the PR-Cre genotype were used as experimental subjects, while wild-type and heterozygous mice served as controls. Transgenic mice were kept in specific pathogen-free (SPF) animal rooms with a 12 h/12 h dark/light cycle. The mice were allowed access to drinking water and food. A Mouse Direct PCR Kit (for genotyping) (B40013, Selleck) was used for genotyping.

Female mice, aged 8 weeks, were euthanized by CO_2_ asphyxiation. Uterine tissues were excised, fixed in formalin overnight, embedded in paraffin, and sectioned for hematoxylin and eosin (HE) staining and immunohistochemistry.

### 2.11 Data analysis

Statistical analysis was performed with GraphPad Prism 5 software (GraphPad Software, Inc., CA, United States). Student’s t-test and one-way ANOVA were used to evaluate the significance of differences; *p* < 0.05 indicated statistical significance (8).

## 3 Results

### 3.1 S100A4 inhibits cell senescence

**Figure 1:**
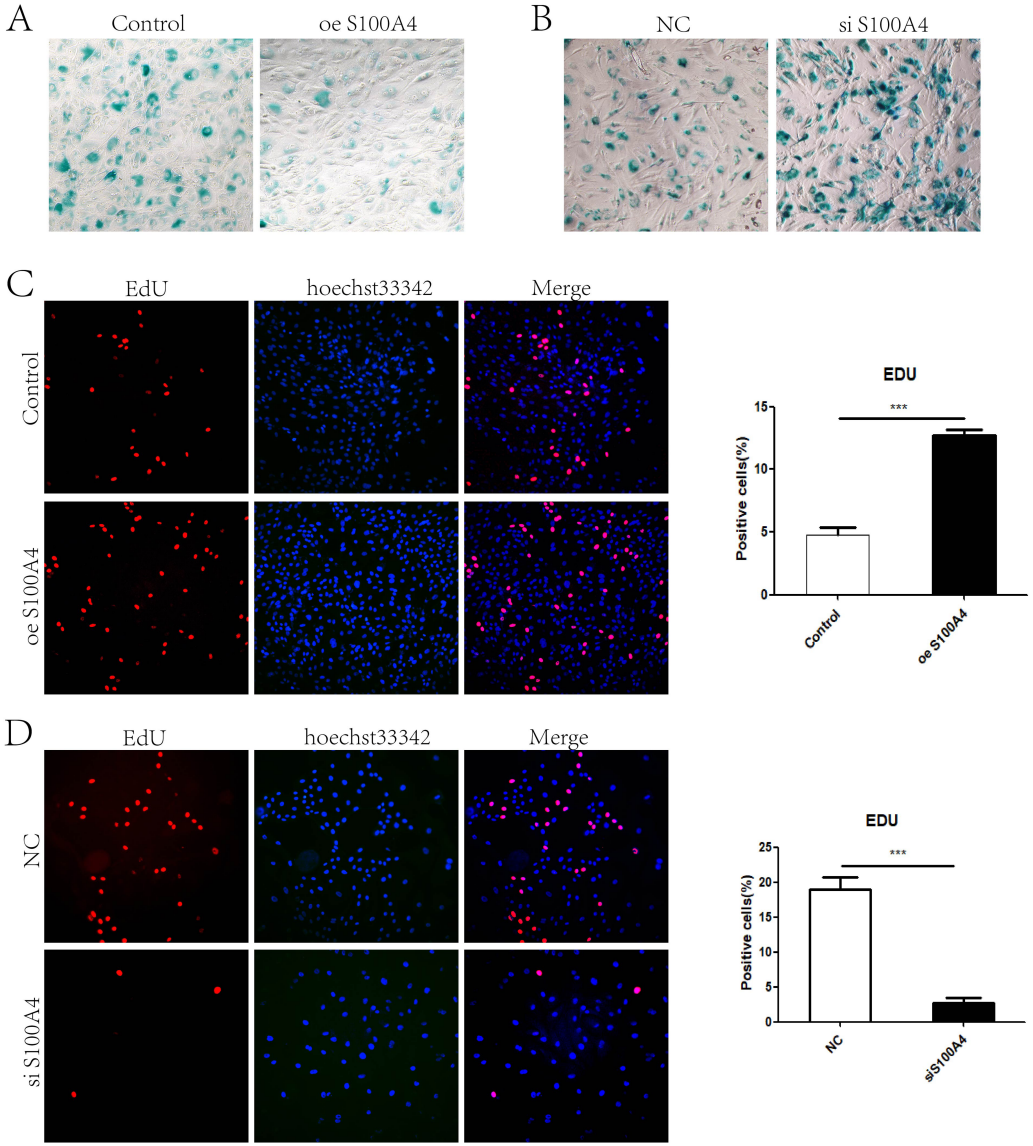
S100A4 inhibits the senescence of sheep endometrial epithelial cells **(A)** Galactosidase staining after S100A4 overexpression. **(B)** Galactosidase staining after S100A4 knockdown. **(C)** EdU staining to detect cell proliferation after S100A4 overexpression. **(D)** EdU staining to detect cell proliferation after S100A4 knockdown.

Previous experiments have shown that S100A4 can promote cell proliferation (8). Considering the cyclical metabolism of endometrial cells and that cell proliferation and senescence are generally opposing processes, we hypothesized that S100A4 might influence the senescence of endometrial cells. Senescence-associated *β*-galactosidase staining can be used to detect cellular senescence. We performed β-galactosidase staining on endometrial cells overexpressing S100A4 for 48 h and on control cells. The results revealed that blue staining was relatively weak in cells overexpressing S100A4, and the proportion of stained cells decreased (Figure 1A). After the cells were transfected with a siRNA targeting S100A4 and stained for *β*-galactosidase after 48 h, the results revealed that, compared with the NC (negative control) group, the S100A4-knockdown group presented an increased number of β-galactosidase-positive cells (Figure 1B). Given that S100A4 promotes cell proliferation, we extended the S100A4 treatment time. After S100A4 was overexpressed or knocked down for 48 h, we performed EdU labeling to detect cell proliferation. The results revealed that the percentage of EdU-positive cells was significantly greater in the S100A4-overexpressing group than in the control group (Figure 1C), whereas the percentage of EdU-positive cells was significantly lower in the S100A4-knockdown group than in the NC group (Figure 1D). These results further indicate that overexpressing S100A4 inhibits cell senescence.

### 3.2 S100A4 inhibits activation of the IL-17 signaling pathway

**Figure 2:**
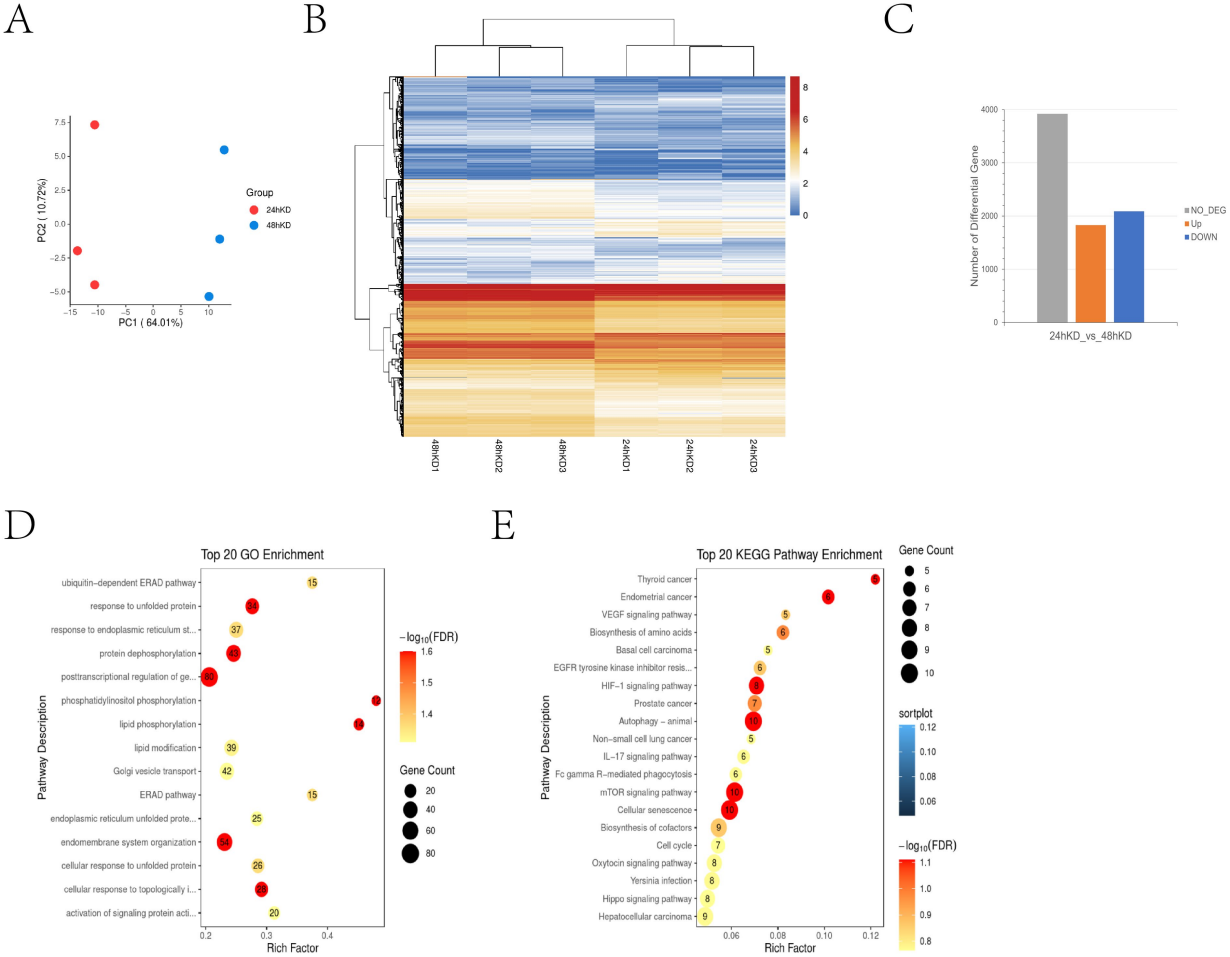
Transcriptome sequencing revealed that S100A4 knockdown can activate the IL-17 signaling pathway **(A)** Principal Component Analysis (PCA) results show significant spatial separation between samples, indicating distinct gene expression profiles after different treatments. For PCA, the first two or three principal components (PC1, PC2) were used to represent the largest variances in gene expression, which allows for clearer differentiation between sample groups. **(B)** Heatmap analysis of gene transcription differences at 24 and 48 h after S100A4 knockdown. The heatmap colors range from blue to red, with blue representing lower expression (log2 fold change) and red representing higher expression. **(C)** Differential gene expression statistics indicate that the number of up-and down-regulated genes (~2,000 each) occurred 24 and 48 h post-S100A4 knockdown. **(D)** GO enrichment analysis highlights key functions, such as post-transcriptional regulation and endomembrane system organization. **(E)** KEGG pathway enrichment identifies IL-17 signaling and pathways related to autophagy and cellular senescence. -log10 (FDR) was used to illustrate the FDR value, with larger values indicating greater statistical significance.

We previously performed transcriptome sequencing 24 h after S100A4 was knocked down and reported that S100A4 can promote cell proliferation by targeting GNAI2–MAPK (8). Because the cell senescence process is relatively slow, we performed transcriptome sequencing 48 h after S100A4 was knocked down to determine how S100A4 regulates cell senescence. Compared with those at 24 h, there were significant changes in cell transcription at 48 h, indicating the formation of a distinct subgroup of cells (Figure 2A). A heatmap generated from the gene transcription data also revealed changes in the expression levels of many genes between 24 and 48 h after S100A4 knockdown (Figure 2B). It is approximately 2000 gene transcripts showed up-regulation and down-regulation respectively (Figure 2C). GO enrichment analysis of the differentially expressed genes revealed enrichment in cellular physiological responses related to senescence, such as the “ubiquitin-dependent ERAD pathway,” “response to unfolded protein,” and “response to endoplasmic reticulum stress” (Figure 2D). These results indicate that the cells initiated the senescence process at the transcriptional level 48 h after S100A4 knockdown. Furthermore, KEGG enrichment analysis revealed significant signaling pathway changes 48 h after S100A4 knockdown compared with 24 h, particularly activation of the IL-17 signaling pathway (Figure 2E). Given that the IL-17 signaling pathway can regulate cell senescence, these results suggest that activating the IL-17 signaling pathway may promote cell senescence following S100A4 knockdown.

### 3.3 S100A4 targets and regulates PPP1CA

**Figure 3:**
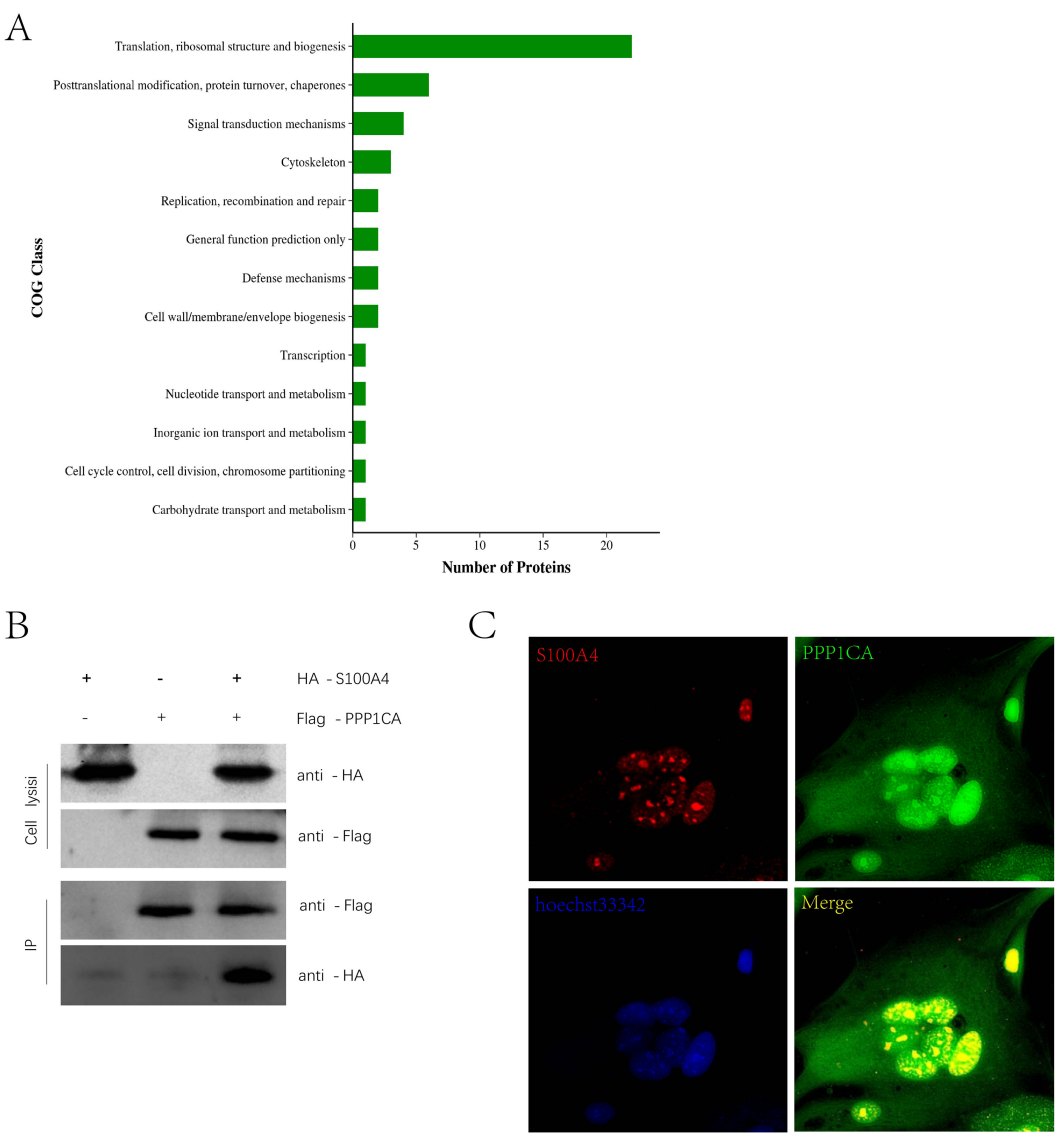
Interaction of S100A4 and PPP1CA **(A)** COG analysis of interacting proteins detected by mass spectrometry after immunoprecipitation of S100A4. **(B)** Immunoprecipitation detection of the interaction between S100A4 and PPP1CA. **(C)** Laser confocal detection of the interaction between S100A4 and PPP1CA.

Previous immunoprecipitation–mass spectrometry studies identified proteins that interact with S100A4 (8). The cluster of orthologous groups (COG) classification of these proteins revealed enrichment in functions related to cell senescence, such as “replication, recombination and repair” and “cell cycle control, cell division, chromosome partitioning” (Figure 3A). Among these proteins, PPP1CA is related to cell senescence. Immunoprecipitation experiments confirmed the interaction between S100A4 and PPP1CA (Figure 3B). Immunofluorescence staining and confocal microscopy revealed that S100A4 (RFP) interacted with PPP1CA (GFP), with the interaction occurring in the cell nucleus (Figure 3C). These results suggest that S100A4 may regulate cell senescence by interacting with PPP1CA.

### 3.4 PPP1CA inhibits cell senescence

**Figure 4:**
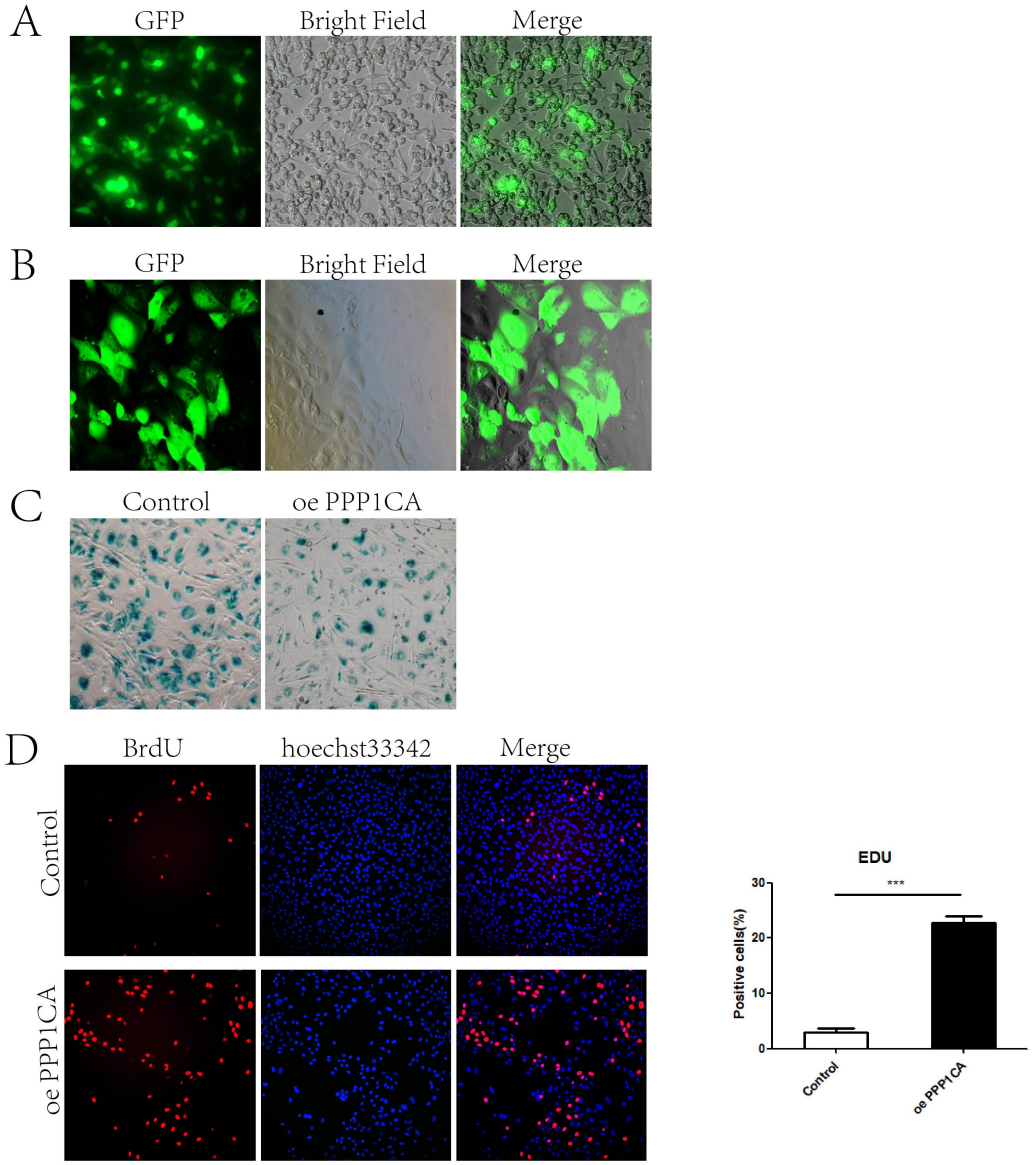
PPP1CA inhibits cell senescence **(A)** 293T cells packaged with lentivirus. **(B)** Lentivirus transduction into sheep endometrial epithelial cells. **(C)** Galactosidase staining after PPP1CA overexpression. **(D)** EdU staining to detect cell proliferation. ***p < 0.001.

To verify whether PPP1CA regulates cell senescence, we constructed a lentiviral vector overexpressing PPP1CA. First, the lentivirus was packaged in 293 T cells (Figure 4A) and then transduced into sheep endometrial cells to overexpress PPP1CA (Figure 4B). *β*-Galactosidase staining 48 h after transduction revealed a reduction in the proportion of positive cells (Figure 4C). PPP1CA overexpression promotes cell proliferation, as demonstrated by EdU staining (Figure 4D). These results suggest that overexpressing PPP1CA can inhibit cell senescence. However, the mechanism by which PPP1CA inhibits the senescence of sheep endometrial cells requires further exploration.

### 3.5 PPP1CA inhibits activation of the IL-17 signaling pathway

**Figure 5:**
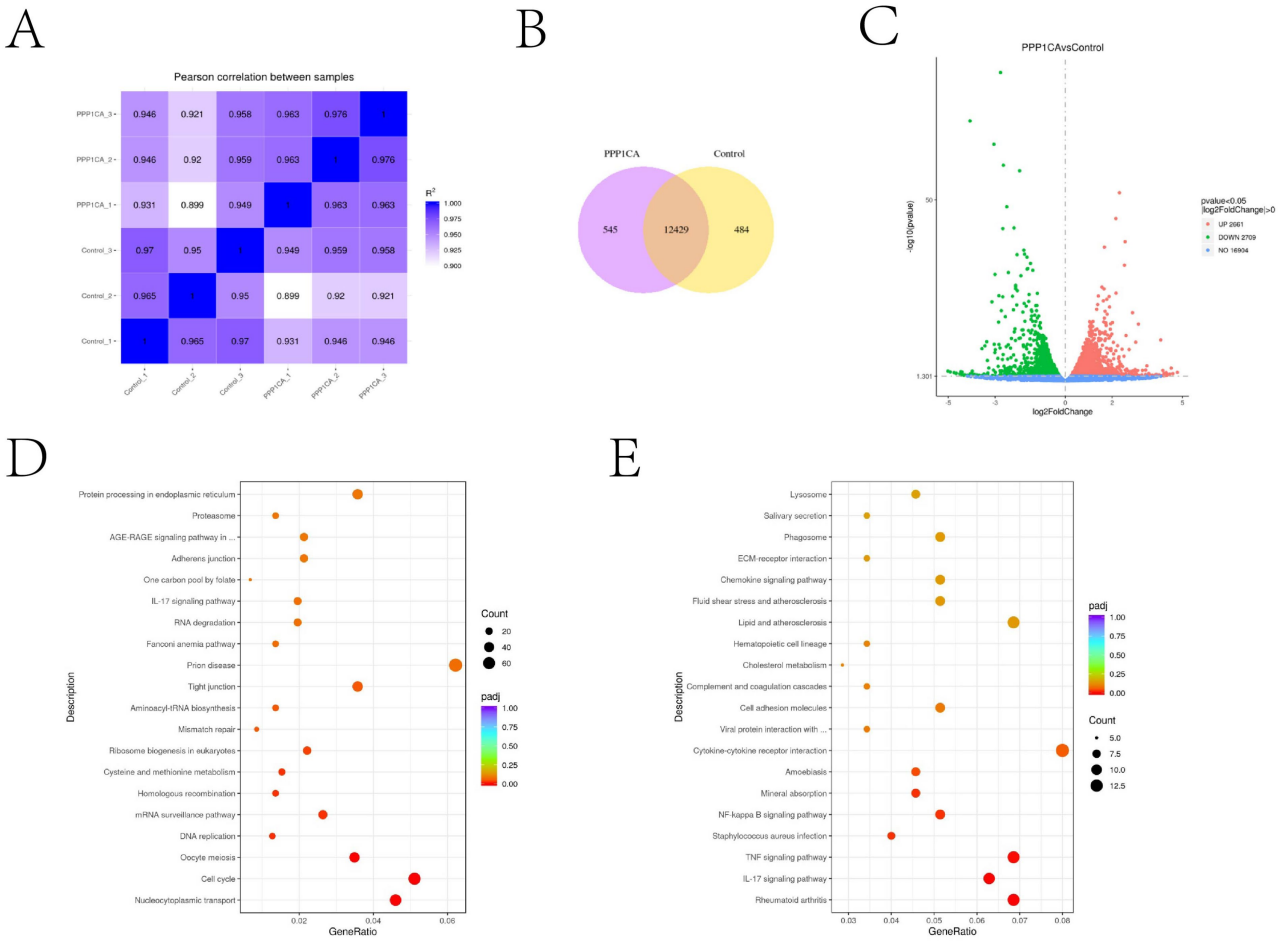
Transcriptome sequencing revealed that PPP1CA can inhibit the IL-17 signaling pathway **(A)** Pearson correlation between samples. **(B)** Changes in the expression levels of specific genes after PPP1CA overexpression. **(C)** Volcano plot showing genes with transcriptional changes after PPP1CA overexpression. -log10 (Pvalue) was used to measure the expression level. The larger the value, the greater the difference in gene expression. **(D)** KEGG enrichment of signaling pathways downregulated after PPP1CA overexpression. **(E)** KEGG enrichment showing differences in signaling pathways after GNAI2 and PPP1CA overexpression. padj (adjusted *p*-value) is a *p*-value that has been corrected to account for the fact that you are performing statistical tests, lower padj values (closer to zero) typically indicate more statistically significant results.

To explore the mechanism by which PPP1CA regulates cell senescence in sheep endometrial cells, we performed transcriptome sequencing on cells overexpressing PPP1CA and control cells. Pearson correlation analysis revealed that the expression profiles of the three PPP1CA-overexpressing groups were all close to 1, indicating stable transcriptional states and reliable sequencing results (Figure 5A). The sequencing results revealed that overexpressing PPP1CA increased the transcription levels of 2,661 genes and decreased the transcription levels of 2,709 genes (Figure 5B). Additionally, 545 genes were newly activated, and 484 genes were silenced upon PPP1CA overexpression (Figure 5C). KEGG enrichment analysis of the downregulated genes revealed pathways such as “protein processing in the endoplasmic reticulum,” “IL-17 signaling pathway,” and “cell cycle” (Figure 5D). Although these pathways are related to cell senescence, they are also associated with cell proliferation. Previous results indicated that GNAI2 could regulate “cell cycle”-related pathways (7). To further identify PPP1CA-specific signaling pathways, we performed differential analysis between genes regulated by GNAI2 and those regulated by PPP1CA (Figure 5E). KEGG clustering analysis revealed enrichment of the IL-17 signaling pathway, suggesting that it may be a key downstream regulatory pathway of PPP1CA.

### 3.6 Inhibition of the IL-17 signaling pathway suppresses cell senescence

**Figure 6:**
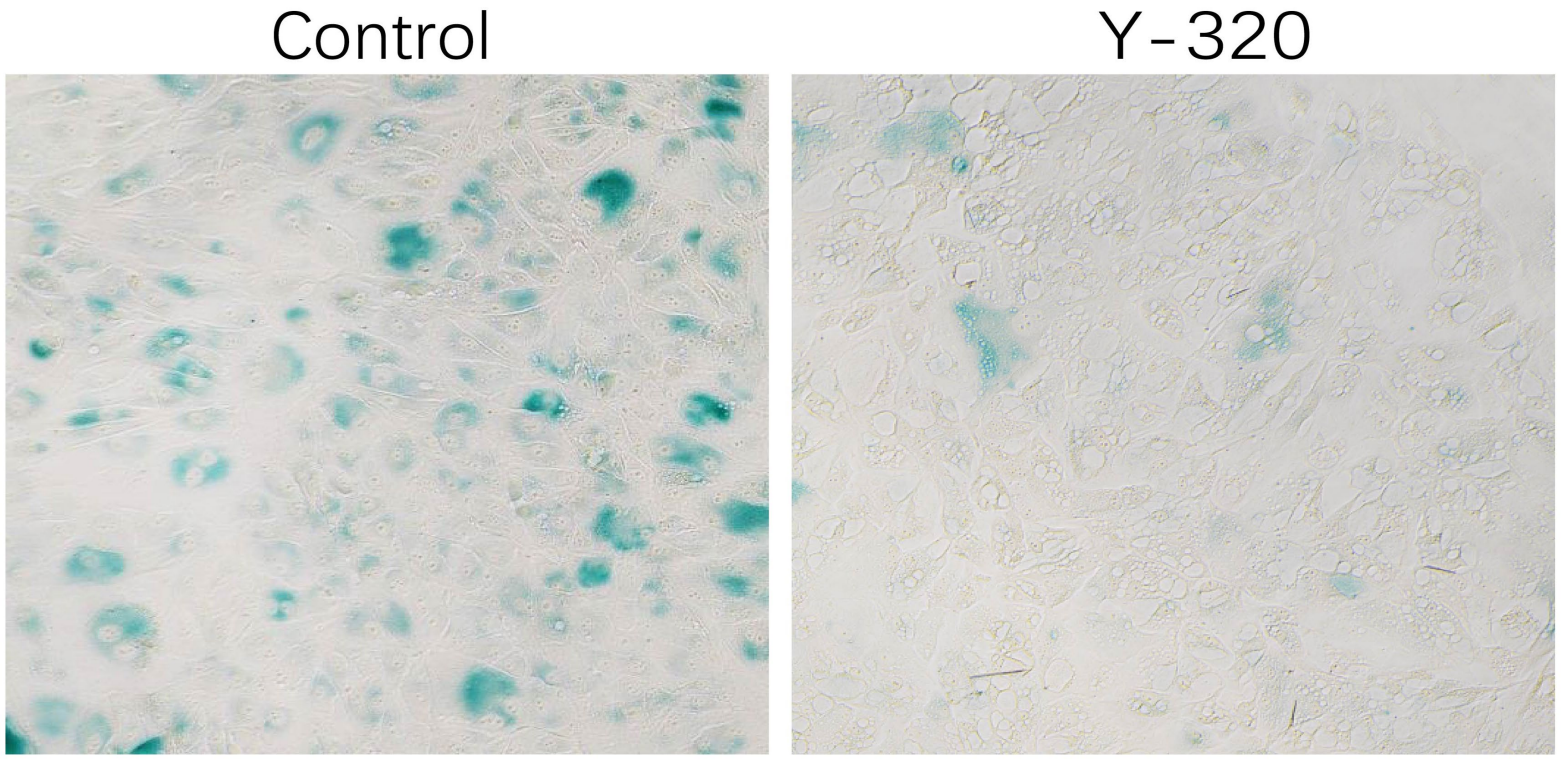
Cell senescence is reduced after treatment with an IL-17 inhibitor.

There is currently no evidence that IL-17 can regulate the proliferation of sheep endometrial cells. To verify whether blocking the IL-17 signaling pathway can inhibit cell senescence, we treated cells with the IL-17 antagonist Y-320 for 48 h. *β*-Galactosidase staining revealed that Y-320 treatment significantly reduced the number of positive cells (Figure 6). In addition, after overexpression of S100A4 and PPP1CA in sheep endometrial cells, decreased IL-17 gene transcription levels were detected (Supplementary Figure 1). These results suggest that the inhibition of the IL-17 signaling pathway resulting from S100A4 and PPP1CA overexpression might be responsible for the reduced senescence of sheep endometrial cells.

### 3.7 *In vivo* experiments show that uterine endometrial cells lacking S100A4 promote senescence

**Figure 7:**
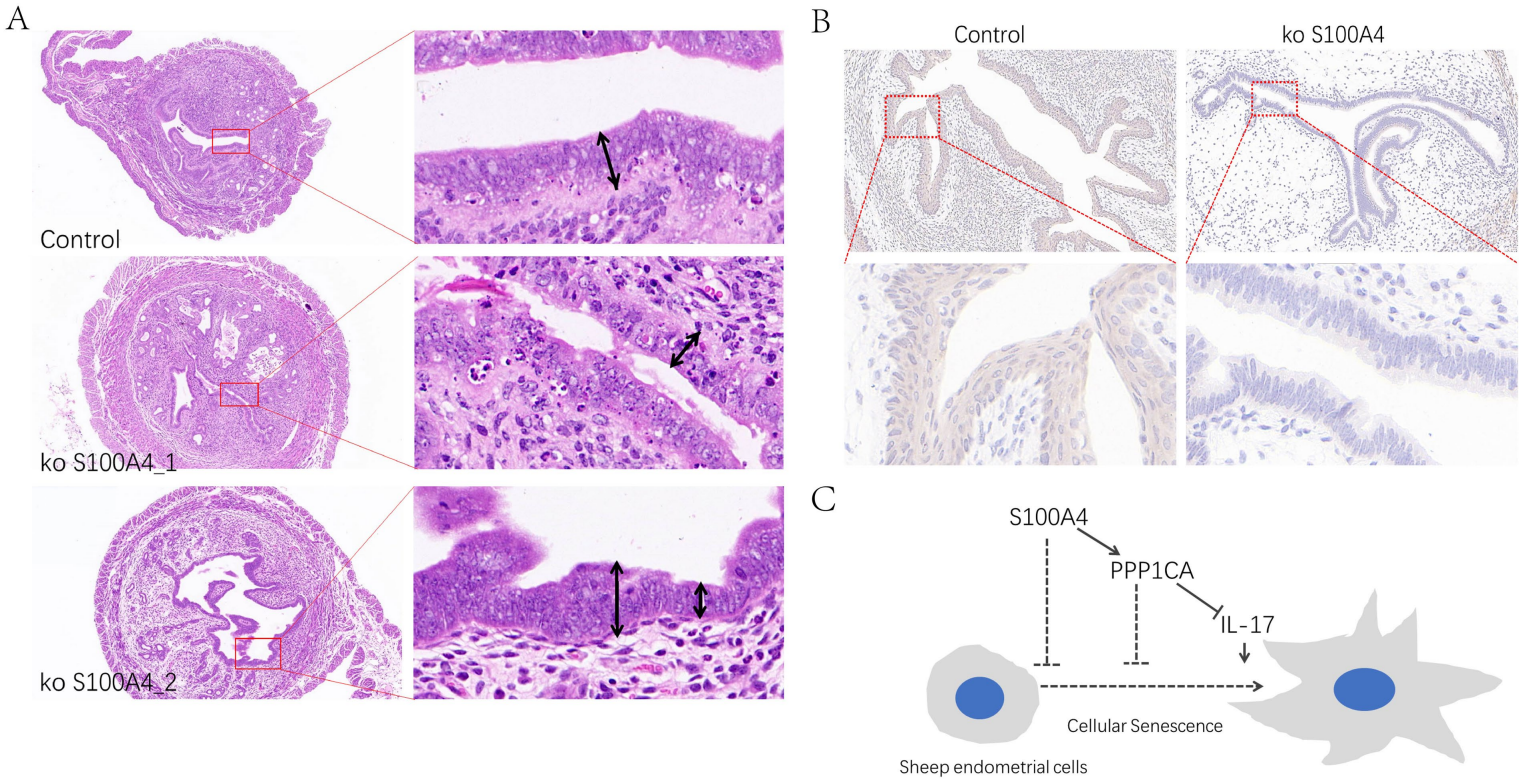
Effects of S100A4 knockout on the mouse endometrial epithelium (A) Effects of HE staining of tissue sections of the endometrium. (B) Immunohistochemical staining of PPP1CA expression in the mouse endometrium. (C) Mechanistic diagram of signaling pathway.

To determine whether S100A4 can regulate PPP1CA *in vivo*, we generated S100A4-deficient mice through breeding (8). HE staining of the endometrium revealed that the epithelial cells in the S100A4-deficient mice were mostly single-layer columnar cells, whereas those in the control group were multilayer epithelial cells. Additionally, the endometrial thickness was uneven and relatively thin in the S100A4-deficient group. Furthermore, nuclear fragmentation and cell death in the epithelial cells of the endometrium in the S100A4-deficient group (Figure 7A) indicate that S100A4 deficiency altered the normal state of endometrial epithelial cells. Immunohistochemical staining revealed reduced PPP1CA expression levels in the endometrial cells of S100A4-deficient mice (Figure 7B). These results suggest that knocking out S100A4 *in vivo* can reduce PPP1CA protein levels lead to endometrial cell damage. Based on these results, it can be speculated that S100A4 and PPP1CA inhibit the senescence of sheep endometrial cells, possibly by inhibiting the activation of the IL-17 signaling pathway (Figure 7C).

## 4 Discussion

In human clinical studies, compared with GnRH agonist protocols, GnRH antagonist protocols for *in vitro* fertilization (IVF) typically result in lower pregnancy rates, with decreased S100P expression, while apoptosis is increased following GnRH antagonist treatment (11). However, the use of GnRH agonists in human EC cell lines reduces cell proliferation (12), a phenomenon that has been confirmed in various cell types (13). During sheep embryo transplantation, GnRH agonists do not facilitate embryo implantation; instead, they inhibit the expression of S100A4, which promotes the proliferation of endometrial epithelial cells (8). In addition to affecting the proliferation of sheep endometrial epithelial cells, GnRH also reduces the number of endometrial cells and induces apoptosis. The final outcome for cells that stop proliferating is often cellular senescence (14). In this study, we found that S100A4 knockdown promotes endometrial epithelial cell senescence, revealing a new mechanism for S100A4’s role in endometrial cell regulation.

The activation of the IL-17 signaling pathway is a major cause of cellular senescence in various cell types. Skin aging is associated with signs of chronic inflammation, and single-cell sequencing has revealed that IL-17 produced by lymphocytes is a cause of skin aging (15). In arthritis, Th17 cells induce fibroblast senescence, and injecting IL-17-neutralizing antibodies into the joints of patients reduces the expression levels of senescence markers (16). Additionally, in degenerative arthritis, the senescence of chondrocytes is significantly linked to IL-17 expression (17). In studies of vascular endothelial dysfunction, IL-17 has been shown to induce endothelial cell senescence (18, 19). This study revealed changes in the IL-17 signaling pathway in S100A4-and PPP1CA-induced senescence, suggesting that IL-17 pathway activation is a significant cause of endometrial cell senescence.

We identified the regulatory molecules S100A4 and PPP1CA, which modulate the IL-17 signaling pathway in endometrial cells. S100A4 expression is significantly decreased in sheep endometrial tissue after GnRH agonist treatment (8). Further *in vitro* experiments revealed that S100A4 knockdown activated the IL-17 pathway. Although no previous reports have linked S100A4 to aging, S100A4 has been extensively studied for its role in promoting the proliferation of breast cancer cells (20, 21), liver cancer cells (22), lung cancer cells (23), prostate cells (24), and endometrial cancer cells (25). In addition to regulating cell proliferation, S100A4 influences immune cell proliferation and mast cell recruitment (26). Single-cell sequencing analysis identified S100A4 as an important immune-suppressing T-cell regulator in glioma (27). S100A4 activates the NF-κB/NLRP3 inflammasome signaling pathway to promote macrophage pyroptosis induced by *Mycobacterium tuberculosis* infection (28). It is also a marker of microglial reactivity, suggesting its role in neuroinflammation (29). These findings imply that S100A4 can regulate cell fate and immune responses. The activation of the IL-17 pathway, which is based on immune response-regulated cellular senescence, indicates that S100A4 has the potential to regulate cellular senescence. Indeed, our experiments confirmed that knocking out S100A4 promotes the senescence of sheep endometrial cells, increasing our understanding of the functional mechanisms of S100A4.

PPP1CA can regulate immune responses, affecting immune cell infiltration and breast cancer tumor cell proliferation (30). In glioma cells, PPP1CA collaborates with KIF18A to regulate cancer cell proliferation (31). In MIO-M1 cells, PPP1CA promotes YAP phosphorylation to regulate cell proliferation (32). In studies of intervertebral disc degeneration, PPP1CA was shown to mediate nucleus pulposus cell senescence (10). Our work revealed the interaction between PPP1CA and S100A4 and verified the role of PPP1CA in sheep endometrial cell senescence. These findings expand the scope of PPP1CA regulation of cellular senescence and elucidate the mechanisms through which PPP1CA is regulated.

## 5 Conclusion

This study revealed that GnRH can regulate endometrial cell senescence, identified the interaction between S100A4 and PPP1CA and described its role in regulating cellular senescence, and revealed the regulatory role of the S100A4/PPP1CA/IL-17 signaling pathway in sheep endometrial cell senescence. These findings provide theoretical guidance for hormone selection during sheep embryo tansfer in clinical settings. Future research on the interaction between different cell types and their impact on sheep endometrial cell senescence will increase the understanding of these mechanisms and aid in identifying regulatory targets to enhance sheep embryo implantation.

## Data Availability

The datasets presented in this study can be found in online repositories. The names of the repository/repositories and accession number(s) can be found at: https://www.ncbi.nlm.nih.gov/, PRJNA1090558; https://www.ncbi.nlm.nih.gov/, PRJNA1090239.
