# Supplementary material for: S100A4 targets PPP1CA/IL-17 to inhibit the senescence of sheep endometrial epithelial cells
Source: Front Vet Sci. 2024 Nov 27;11:1466482. doi: 10.3389/fvets.2024.1466482 (PMC11633043; doi:10.3389/fvets.2024.1466482)
Supplement: Supplementary file 1 [file Presentation_1.PPTX]

## Slide 1
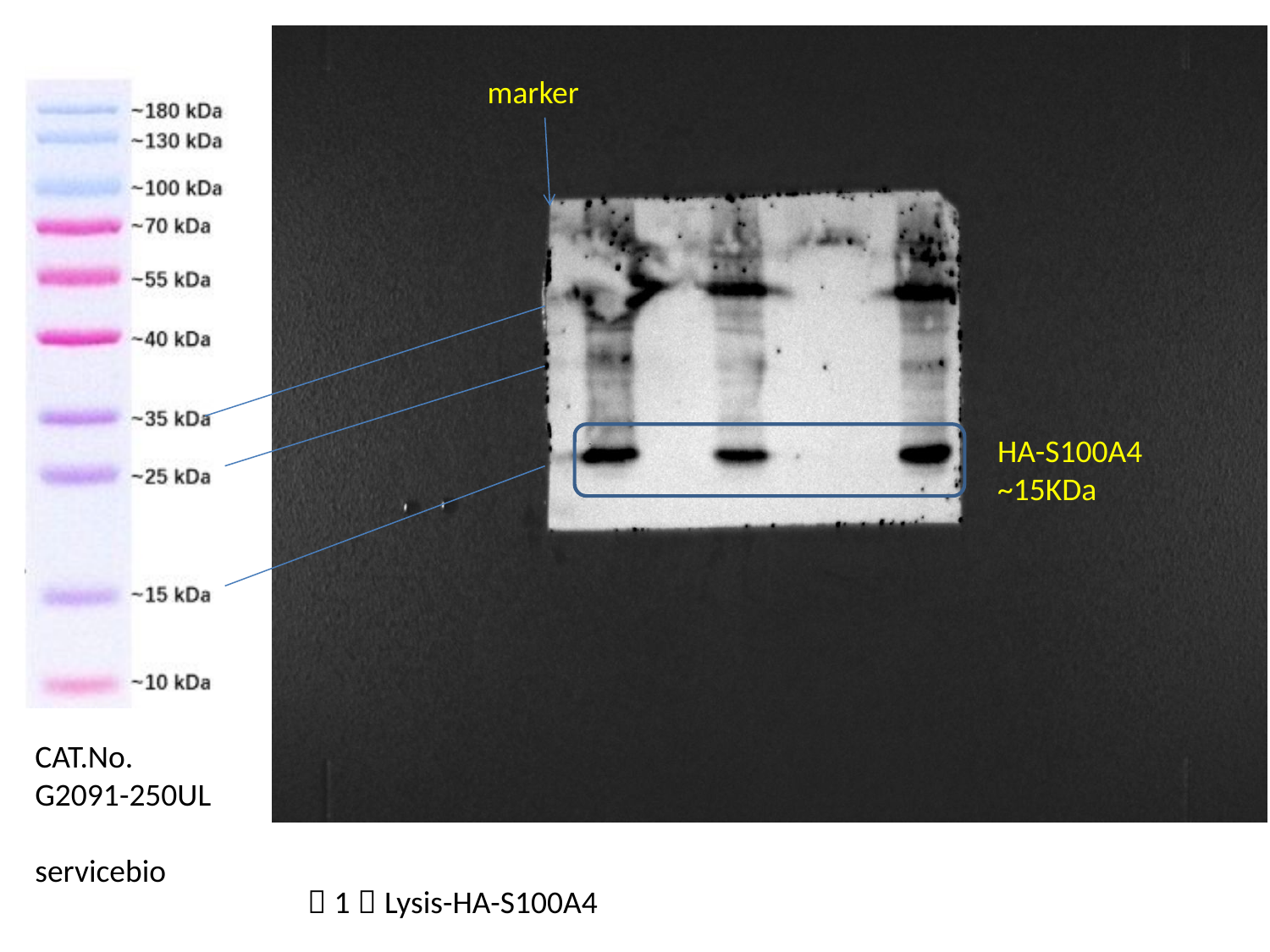

marker
CAT.No.
G2091-250UL
servicebio
（1）Lysis-HA-S100A4
HA-S100A4
~15KDa

## Slide 2
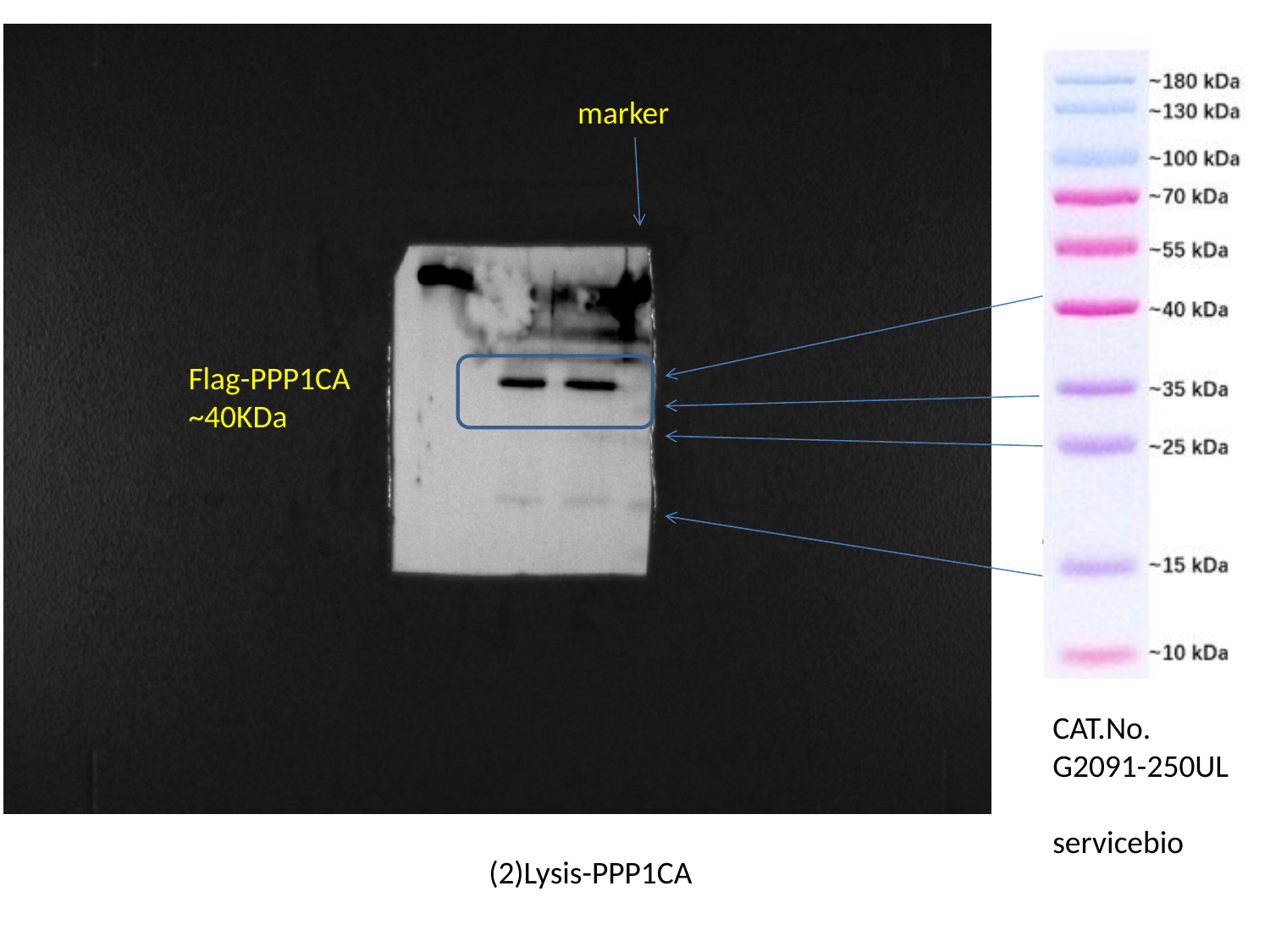

marker
Flag-PPP1CA
~40KDa
CAT.No.
G2091-250UL
servicebio
(2)Lysis-PPP1CA

## Slide 3
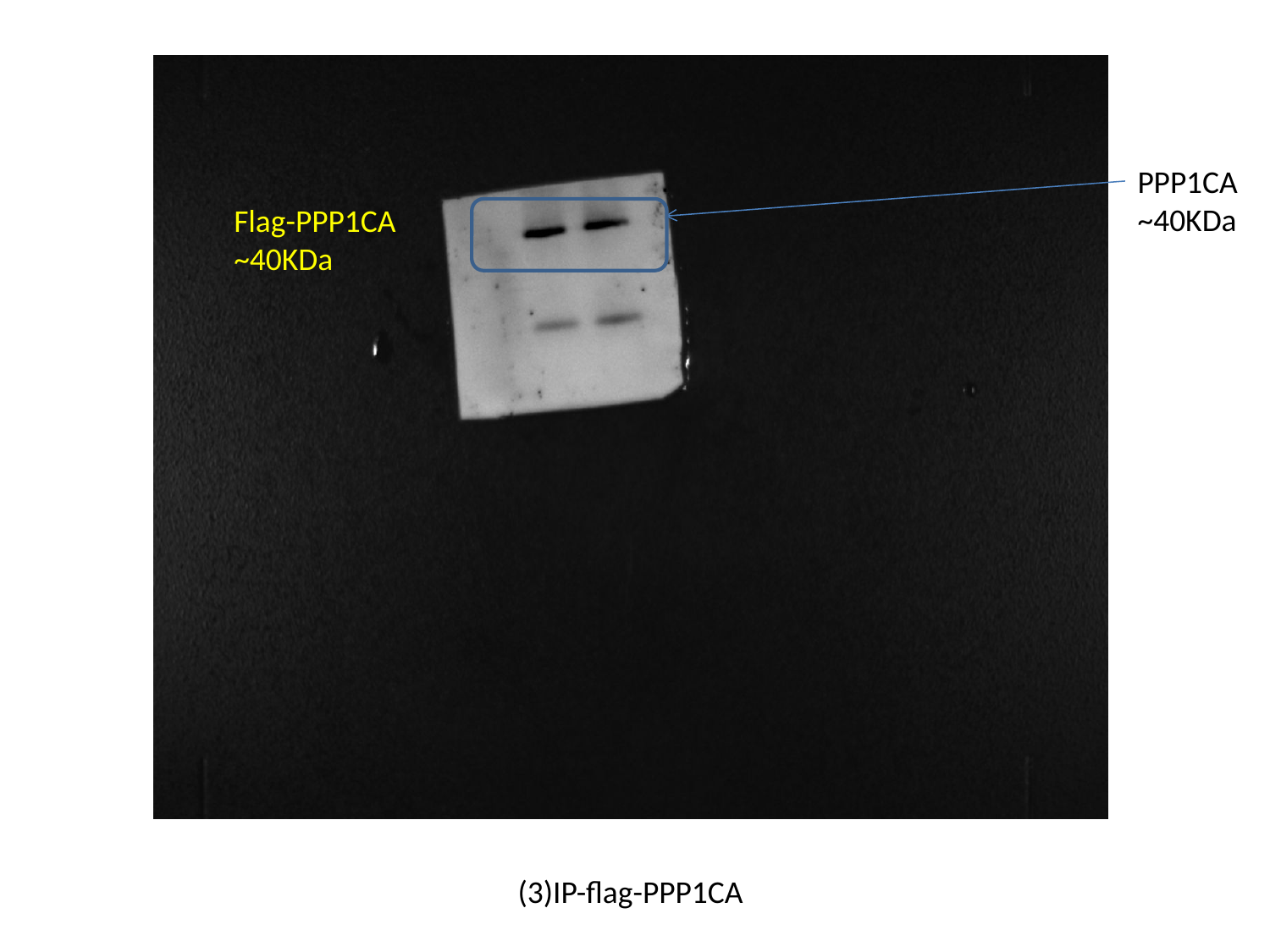

PPP1CA
~40KDa
Flag-PPP1CA
~40KDa
(3)IP-flag-PPP1CA

## Slide 4
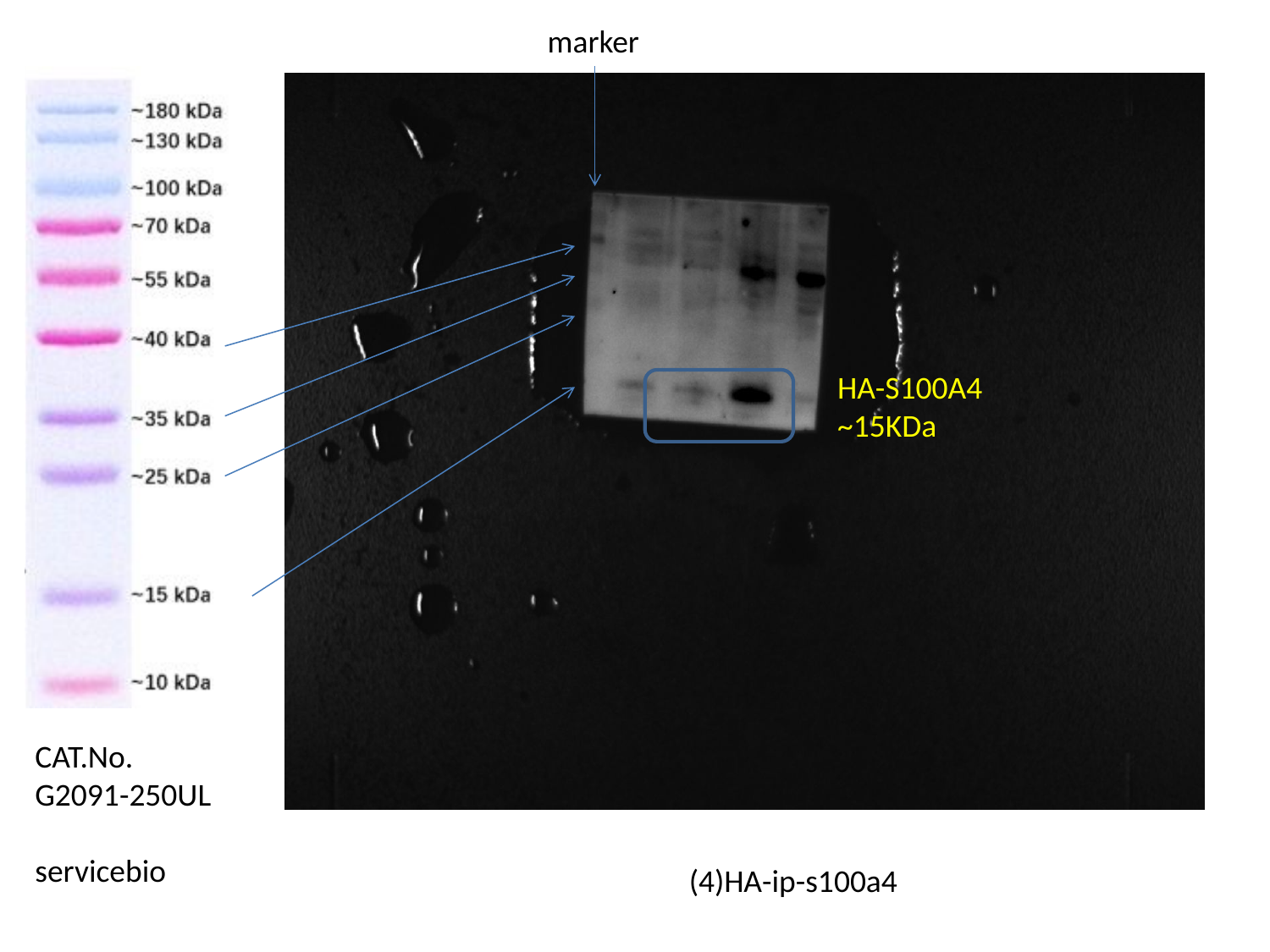

marker
HA-S100A4
~15KDa
CAT.No.
G2091-250UL
servicebio
(4)HA-ip-s100a4
