# Supplementary material for: S100A4 targets PPP1CA/IL-17 to inhibit the senescence of sheep endometrial epithelial cells
Source: Front Vet Sci. 2024 Nov 27;11:1466482. doi: 10.3389/fvets.2024.1466482 (PMC11633043; doi:10.3389/fvets.2024.1466482)
Supplement: Supplementary file 2 [file Data_Sheet_1.PDF]

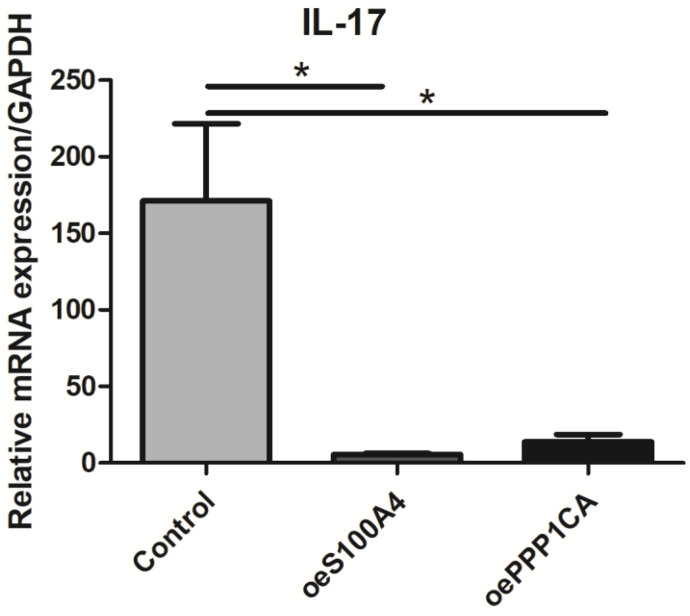

Supplementary Picture 1

RT-qPCR was used to detect the effect of overexpression of S100A4 and PPP1CA on the transcription level of IL-17 gene. *P＜0.05.
