# Supplementary material for: S100A4 targets PPP1CA/IL-17 to inhibit the senescence of sheep endometrial epithelial cells
Source: Front Vet Sci. 2024 Nov 27;11:1466482. doi: 10.3389/fvets.2024.1466482 (PMC11633043; doi:10.3389/fvets.2024.1466482)
Supplement: Supplementary file 4 [file Data_Sheet_3.PDF]

SERVICE TYPE: DNA CONSTRUCT	
**Certificate of Analysis**	
Project ID:	GS1-22050290

Construct Information

**Gene Name**	PPP1CA-XM_012102181.4-3FLAG	**Gene Length**	1077 bp
**Cloning Vector**	pCDH-CMV-MCS-EF1-CopGFP-T2A-Puro	**Cloning Strategy**	NheI EcoRI
**Construct Resistant**	Amp	**Construct ID**	Y19788-150817

QC Items

QC Items	Method	Specifications	Results
Target Sequence	Sequence alignment	Sequencing results are consistent to the confirmed sequence.	Pass
Vector Sequence	Sequence alignment	20bp flanking sequences of the vector are correct.	NA
Reading Frame	Sequence alignment	Frame is correct and consistent to the client's requirement.	Pass
Fragment Size	Restriction Digests	The size of inserted fragment is right and free of any contaminated bands.	Pass
Quality/Quantity	UV spectrophotometry	Miniprep: OD260/280=1.7~2.0<br>4μg/tube, 1 tube, Lyophilized	Pass
Appearance	Visual inspection	Clear and free of foreign particles.	Pass
Customized Test	NA	NA	NA

Restriction Digestion Map

	M	1	2	

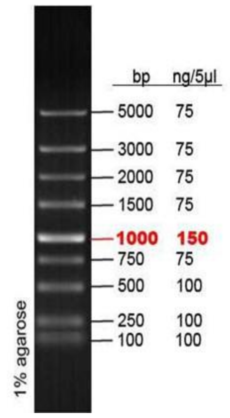

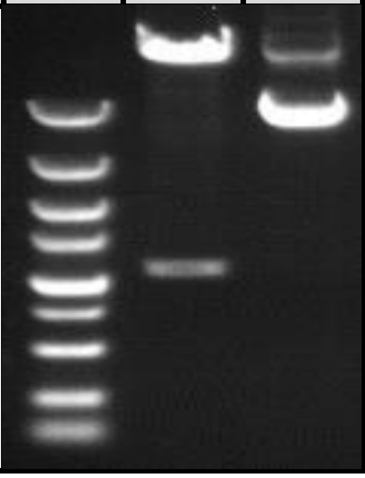

Lane M: DNA Marker

Lane 1: Plasmid digested by NheI EcoRI

Lane 2: Plasmid DNA

Date: 2022/6/3
